# Relative Tail Pressure with Subadditive Potentials

**DOI:** 10.3390/e21121178

**Published:** 2019-11-29

**Authors:** Xianfeng Ma, Baoli Liu, Ercai Chen

**Affiliations:** 1 Department of Mathematics, East China University of Science and Technology, Shanghai 200237, China; Y30170139@mail.ecust.edu.cn; 2 School of Mathematical Science, Nanjing Normal University, Nanjing 210097, China; ecchen@njnu.edu.cn

**Keywords:** relative topological tail pressure, subadditive potentials, random covers, variational principle

## Abstract

The relative tail pressure with subadditive potentials is introduced via open random covers for continuous bundle random transformations. A variational principle is established and the defined pressure turns out to be invariant under the principal extension.

## 1. Introduction

The topological pressure with additive potential was introduced by Ruelle and Walters [1,2]. The topological pressure for subadditive sequence of continuous potentials was introduced by Falconer [3] on mixing repellers. Cao et al. [4] considered it on compact systems and established its corresponding variational principle. Ledrappier and Walters [5] introduced a relative version of topological pressure in the field of the relativized ergodic theory. Bogenschütz [6] defined the topological pressure for random transformations in the stationary case. Kifer [7] proposed the notion of topological pressure for continuous bundle random transformations and established its variational principle. The topological pressure play a fundamental role in statistical mechanics, dimension theory [8,9,10,11,12,13] and in the study of complex properties of a random dynamical system [14,15,16,17,18].

The topological pressure with zero potential reduces to the classical topological entropy. For the purpose of measuring the local complexity of compact dynamical systems at arbitrary small scales, Misiurewicz introduced topological tail entropy [19] for continuous transformations. Downarowicz [20,21] established a maximal entropy principle for the topological tail entropy for homeomorphism. In terms of the essential partitions, Burguet [22] proved the principle for continuous transformations. Ledrappier [23] investigated the defect of upper semi-continuity of the metric entropy on the square of compact systems, presented a maximal entropy principle relating it with the topological tail entropy, and showed that topological tail entropy is invariant under the principal extension. The relative version of the tail entropy for continuous bundle random transformations was introduced by Kifer and Weiss [24] and they deduced the consistence of the two entropy notions defined by open covers and spanning subsets.

Ma et al. set up a relative tail maximal entropy principle [25] and a tail variational principle [26] for continuous random transformations by introducing the relative tail entropy and pressure via open random covers, both of the two quantities are proved to be conserved under principal extensions. The notions defined there, via random covers, can enable ones to investigate different fibers under a natural but more complex cover way. For the nonadditive potentials case, the nonadditive thermodynamic formalisms are the powerful tools for the theory of multifractal analysis [12,27,28,29]. A natural question arises whether the tail variational principle still holds for the relative tail pressure associated with a sequence of subadditive random continuous potentials and whether this kind of tail pressure can be maintained by the action of principal extensions.

In this paper we introduce the relative tail pressure with subadditive potentials for continuous bundle random transformations via open random sets. The notion is a little different from that developed before [24,30] for the same cover of the fibers, which could make us consider various covers on different fibers. We investigate the product continuous bundle random dynamical system (RDS) generated by a given continuous bundle RDS and another continuous bundle RDS over the same probability space. A variational inequality is obtained for the relative tail pressure with subadditive potentials, which shows that the pressure of given continuous bundle RDS is an upper bound of the defect of the upper semi-continuity of the relative entropy and Lyapunov exponent of subadditive potentials in the product continuous bundle RDS. For the self-product of the given continuous bundle RDS, we establish a variational principle for the defined pressure by constructing a maximal invariant measure for the product continuous bundle RDS to ensure that the relative tail pressure may be attained. As for the trivial measure space, the relative tail pressure with the zero potential is just the topological tail entropy defined in Reference [19] and the variational principle is the deterministic version of maximal entropy principle deduced by Ledrappier [23]. It turns out that from this variational principle that the relative tail pressure with subadditive potentials is an invariant in the sense of the principal extension. The method we adopt is still in the framework of Misiurewicz’s elegant proof [31].

Organization of the paper is as follows?We recall some basics of the relativized ergodic theory in Section 2. The relative tail pressure with subadditive potentials is introduced in term of open random covers in Section 3. In Section 4, we give the power rule and a variational inequality for the relative tail pressure in the general product RDSs. In Section 5, we state and prove the variational principle in the self-product RDSs for the relative tail pressure with the subadditive potentials and show that the defined pressure can be conserved under the consideration of the principal extension.

## 2. Relative Entropy

In this section, we recall some basic notions of the relative measure-theoretic entropies for bundle random transformations [24,26]. For a general theory of random dynamical systems, we refer to [24,32,33].

Let (Ω,F,P) be a probability space which is complete countably generated and ϑ be a P-preserving transformation of this space. Let *X* be a compact metric space and B be its Borel σ-algebra. Let E be subset of Ω×X which is measurable under the product σ-algebra F×B and assume that the fibers $E_{\omega }=\left\{x\in X:\left(\omega ,x\right)\in E\right\}$ are compact subsets of *X*. A continuous bundle random dynamical system (RDS) *T* over (Ω,F,P,ϑ) is generated by the mappings $T_{\omega }:E_{\omega }\to E_{\vartheta \omega }$ so that the map $\left(\omega ,x\right)\to T_{\omega }x$ is measurable and the map $x\to T_{\omega }x$ is continuous for P-almost all (a.a.) ω. The family $\left\{T_{\omega }:\omega \in \Omega \right\}$ is called a random transformation and each $T_{\omega }$ maps the fiber $E_{\omega }$ to $E_{\vartheta \omega }$. The map Θ:E→E defined by $\Theta \left(\omega ,x\right)=\left(\vartheta \omega ,T_{\omega }x\right)$ is called the skew product transformation. Notice that $\Theta^{n}\left(\omega ,x\right)=\left(\vartheta^{n}\omega ,T_{\omega }^{n}x\right)$, where $T_{\omega }^{n}=T_{\vartheta^{n-1}\omega }\circ \cdots T_{\vartheta \omega }\circ T_{\omega }$ for n≥0 and $T_{\omega }^{0}=id$.

Let $P_{P}\left(\Omega \times X\right)$ be the space of probability measures on Ω×X with the marginal P on Ω and $P_{P}\left(E\right)=\left\{\mu \in P_{P}\left(\Omega \times X\right):\mu \left(E\right)=1\right\}$. Denote by $I_{P}\left(E\right)$ the space of all Θ-invariant measures in $P_{P}\left(E\right)$.

Let $\mu \in I_{P}\left(E\right)$ and T be a sub-σ-algebra of F×B which is restricted on E and satisfies $\Theta^{-1}T\subset T$. Let R be a finite or countable measurable partition of E, the relative entropy $h_{\mu }\left(R|T\right)$ of Θ is defined as

$$h_{\mu }\left(R|T\right)=\lim_{n\to \infty }\frac{1}{n}H_{\mu }\left(R^{\left(n\right)}|T\right)=\underset{n}{\inf}\frac{1}{n}H_{\mu }\left(R^{\left(n\right)}|T\right),$$

where $H_{\mu }\left(R^{\left(n\right)}|T\right)$ is the conditional entropy of $R^{\left(n\right)}$ given σ-algebra T and $R^{\left(n\right)}={⋁}_{i=0}^{n-1}{\left(\Theta^{i}\right)}^{-1}R$.

The relative entropy of Θ is defined by the formula

$$h_{\mu }\left(\Theta |T\right)=\underset{R}{\sup}h_{\mu }\left(R|T\right),$$

where the supremum is taken over all finite or countable measurable partitions R of E with finite conditional entropy $H_{\mu }\left(R|T\right)<\infty$. The defect of upper semi-continuity of the relative entropy $h_{\mu }(\Theta |T$) is defined on $I_{P}\left(E\right)$ as

$$h_{m}^{*}\left(\Theta |T\right)=\left\{\begin{array}{cc} \underset{\mu \to m}{lim sup}h_{\mu }\left(\Theta |T\right)-h_{m}\left(\Theta |T\right),\; & \mathrm{if}\;\;h_{m}\left(\Theta |T\right)<\infty ,\\ \infty , & \mathrm{otherwise}. \end{array}\right.$$

## 3. Relative Tail Pressure with Subadditive Potentials

Let $\Phi ={\left\{f_{n}\right\}}_{n=1}^{\infty }$ be a sequence of random continuous functions on E in $L_{E}^{1}\left(\Omega ,C\left(X\right)\right)$(see Reference [34] for the detail). Φ is called subadditive if for any (ω,x)∈E and m,n∈N,

$$f_{n+m}\left(\omega ,x\right)\leq f_{n}\left(\omega ,x\right)+f_{m}\left(\Theta^{n}\left(\omega ,x\right)\right).$$

For any Θ-invariant measure μ, denote

$$\Phi^{*}\left(\mu \right)=\lim_{n\to \infty }\frac{1}{n}\int f_{n}\;d\mu .$$

The existence of the limit follows from the well-known subadditive argument. $\Phi^{*}\left(\mu \right)$ is called the Lyapunov exponent of Φ with respect to μ. Denote by $\Phi^{k}={\left\{f_{kn}\right\}}_{n=1}^{\infty }$ for any k∈N, then ${\left(\Phi^{k}\right)}^{*}\left(\mu \right)=k\Phi^{*}\left(\mu \right)$.

The map $Q:\Omega \to 2^{X}$ is called a (closed) random set if *Q* is measurable, where $2^{X}$ denotes the space of the (closed) subsets of *X*. The map $U:\Omega \to 2^{X}$ is called an open random set if its complement $U^{c}$ is closed. Let Q be a finite or countable family of random sets {Q} and denote Q(ω)={x:(ω,x)∈Q}. Q is called a random cover of E if $E_{\omega }={⋃}_{Q\in Q}Q\left(\omega \right)$ for all ω∈Ω. U is called an open random cover if all random set *U* in U are open. Let Q(ω)={Q(ω)}. We will denote by P(E), U(E) the set of random covers, open random covers, respectively. A random cover R is said to be finer than another random cover Q, written as R≻Q, if each element of R is a subset of some element of Q.

For each R∈P(E) and any non-empty set E⊂E, denote

$$P_{\Theta }^{\Phi }\left(\omega ,n,R,E\right)=\underset{\eta }{\inf}\left\{\sum_{S\in \eta }\underset{x\in S(\omega )\cap E(\omega ))}{\sup}e^{f_{n}\left(\omega ,x\right)}\right\},$$

where η belongs to the set of all random subcover of $R^{\left(n\right)}$. For R,Q∈P(E), let

$$P_{\Theta }^{\Phi }\left(\omega ,n,R,Q\right)=\max_{Q\in Q^{\left(n\right)}}P_{\Theta }^{\Phi }\left(\omega ,n,R,Q\right)\}.$$

For each ω, a standard argument shows that the sequence $\log P_{\Theta }^{\Phi }\left(\omega ,n,R,Q\right)$ is subadditive.

By replacing the function $S_{n}f$ in Lemma 3.1 of Reference [26] with $f_{n}$, one can easily get the following Lemma, which provides the basic measurable property needed. In fact, for any measurable function *g* on E, this result also holds.

**Lemma** **1.** *The map $\omega \to P_{\Theta }^{\Phi }\left(\omega ,n,U,Q\right)$ from *Ω* to R is measurable for each U∈U(E) and Q∈P(E).*

The limit

$$P_{\Theta }^{\Phi }\left(\omega ,U,Q\right)=\lim_{n\to \infty }\frac{1}{n}\log P_{\Theta }^{\Phi }\left(\omega ,n,U,Q\right)$$

P-a.s. exists, which follows from the classical subadditive ergodic theorem (see Reference [33,35]). Let

$$\pi_{\Theta }^{\Phi }\left(U,Q\right)=\lim_{n\to \infty }\frac{1}{n}\int \log P_{\Theta }^{\Phi }\left(\omega ,n,U,Q\right)dP=\int P_{\Theta }^{\Phi }\left(\omega ,U,Q\right)dP.$$

Notice that $\pi_{\Theta }^{\Phi }\left(U,Q\right)$ increase in U, a limit (finite or infinite) exists over the directed set U(E),

$$\pi_{\Theta }^{\Phi }\left(Q\right)=\lim_{U\in U(E)}\pi_{\Theta }^{\Phi }\left(U,Q\right)=\underset{U\in U(E)}{\sup}\pi_{\Theta }^{\Phi }\left(U,Q\right).$$

$\pi_{\Theta }^{\Phi }\left(Q\right)$ is said to be the relative conditional pressure of Θ with subadditive potentials Φ for random cover Q. For the trivial Q, $\pi_{\Theta }^{\Phi }\left(Q\right)$ will be simply written as $\pi_{\Theta }\left(\Phi \right)$. Since $\pi_{\Theta }^{\Phi }\left(Q\right)$ decrease in Q, another limit exists over P(E),

$$\pi_{\Theta }^{*}\left(\Phi \right)=\lim_{Q\in P(E)}\pi_{\Theta }^{\Phi }\left(Q\right)=\underset{Q\in P(E)}{\inf}\pi_{\Theta }^{\Phi }\left(Q\right),$$

$\pi_{\Theta }^{*}\left(\Phi \right)$ is said to be the relative tail pressure of Θ with subadditive potentials Φ. Obviously $\pi_{\Theta }^{*}\left(\Phi \right)\leq \pi_{\Theta }\left(\Phi \right)$.

## 4. Variational Inequality for Relative Tail Pressure

In this section we consider the relationship between the relative tail pressure, Lyapunov exponent with subadditive potentials and the relative entropy over the measurable subset of the product space Ω×Y×X.

We first give the power rules for the relative conditional pressure and relative tail pressure with subadditive potentials in the original continuous bundle RDS.

**Proposition** **1.** *Let *Θ* be a skew product transformation, *Φ* be subadditive and Q∈P(E). Then $\pi_{\Theta^{m}}^{\Phi^{m}}\left(Q^{\left(m\right)}\right)=m\pi_{\Theta }^{\Phi }\left(Q\right)$ for each m∈N.*

**Proof.**  Let U∈U(E). Notice that

$$\overset{n-1}{\underset{j=0}{⋁}}{\left(\Theta^{mj}\right)}^{-1}\left(\overset{m-1}{\underset{i=0}{⋁}}{\left(\Theta^{i}\right)}^{-1}U\right)=\overset{nm-1}{\underset{i=0}{⋁}}{\left(\Theta^{i}\right)}^{-1}U,$$

and then

$$P_{\Theta^{m}}^{\Phi^{m}}\left(\omega ,n,U^{\left(m\right)},Q^{\left(m\right)}\right)=P_{\Theta }^{\Phi }\left(\omega ,nm,U,Q\right).$$

Clearly,

$$\begin{array}{cc} \pi_{\Theta^{m}}^{\Phi^{m}}\left(U^{\left(m\right)},Q^{\left(m\right)}\right)\; & =\lim_{n\to \infty }\frac{1}{n}\int \log P_{\Theta }^{\Phi }\left(\omega ,nm,U,Q\right)dP\\ \; & =\lim_{n\to \infty }m\frac{1}{nm}\int \log P_{\Theta }^{\Phi }\left(\omega ,nm,U,Q\right)dP\\ \; & =m\pi_{\Theta }^{\Phi }\left(U,Q\right). \end{array}$$

Then

$$m\pi_{\Theta }^{\Phi }\left(Q\right)=\underset{U\in U(E)}{\sup}\pi_{\Theta^{m}}^{\Phi^{m}}\left(U^{\left(m\right)},Q^{\left(m\right)}\right)\leq \pi_{\Theta^{m}}^{\Phi^{m}}\left(Q^{\left(m\right)}\right).$$

Since $U≺U^{\left(m\right)},$ then

$$\pi_{\Theta^{m}}^{\Phi^{m}}\left(U,Q^{\left(m\right)}\right)\leq \pi_{\Theta^{m}}^{\Phi^{m}}\left(U^{\left(m\right)},Q^{\left(m\right)}\right)=m\pi_{\Theta }^{\Phi }\left(U,Q\right).$$

Thus $\pi_{\Theta^{m}}^{\Phi^{m}}\left(Q^{\left(m\right)}\right)\leq m\pi_{\Theta }^{\Phi }\left(Q\right)$ and the result holds. □

**Proposition** **2.** Let *Θ* be a skew product transformation and *Φ* be subadditive. Then $\pi_{\Theta^{m}}^{*}\left(\Phi^{m}\right)=m\pi_{\Theta }^{*}\left(\Phi \right)$ for each m∈N.

**Proof.**  By Proposition 1,

$$\underset{Q}{\inf}\pi_{\Theta^{m}}^{\Phi^{m}}\left(Q^{\left(m\right)}\right)=\underset{Q}{\inf}m\pi_{\Theta }^{\Phi }\left(Q\right)=m\pi_{\Theta }^{*}\left(\Phi \right),$$

where Q belongs to the set of all random covers of E. Then $\pi_{\Theta^{m}}^{*}\left(\Phi^{m}\right)\leq m\pi_{\Theta }^{*}\left(\Phi \right).$

Since $Q≺Q^{\left(m\right)}$ for each Q∈P(E), then $\pi_{\Theta^{m}}^{\Phi^{m}}\left(Q\right)\geq \pi_{\Theta^{m}}^{\Phi^{m}}\left(Q^{\left(m\right)}\right).$ Then $\pi_{\Theta^{m}}^{*}\left(\Phi^{m}\right)\geq m\pi_{\Theta }^{*}\left(\Phi \right)$ by taking infimum over all Q∈P(E) on this inequality. □

Consider another compact space (Y,C). Denote by G a measurable subset of Ω×Y satisfying that the fibers $G_{\omega }$ are compact. The continuous bundle RDS *S* over (Ω,F,P,ϑ) and the skew product transformation Λ on G can be defined similarly as in Section 2.

**Definition** **1.** *A continuous bundle RDS T is called a factor of another continuous bundle RDS S if a family of continuous surjective maps $\pi_{\omega }:G_{\omega }\to E_{\omega }$ exists, which satisfies the map $\left(\omega ,y\right)\to \pi_{\omega }y$ being measurable and $\pi_{\vartheta \omega }S_{\omega }=T_{\omega }\pi_{\omega }$. The factor transformation π from G to E is defined as $\pi \left(\omega ,y\right)=\left(\omega ,\pi_{\omega }y\right)$ and the skew product system (E,Θ) is said to be a factor of the skew product system (G,Λ).*

We now take up the consideration of the measurable subset H based on G and E with the product σ-algebra F×C×B. Denote by $H=\{\left(\omega ,y,x\right):y\in G_{\omega },x\in E_{\omega }\}$ and set $H_{\omega }=\left\{\left(y,x\right):\left(\omega ,y,x\right)\in H\right\}$. The continuous bundle RDS S×T over (Ω,F,P,ϑ) can be defined as usual by the maps ${\left(S\times T\right)}_{\omega }:H_{\omega }\to H_{\vartheta \omega }$, which requires $\left(\omega ,y,x\right)\to \left(S_{\omega }y,T_{\omega }x\right)$ being measurable and $\left(y,x\right)\to \left(S_{\omega }y,T_{\omega }x\right)$ being continuous in (y,x) for P-a.a. ω. The skew product transformation Γ is defined as $\Gamma \left(\omega ,y,x\right)=\left(\vartheta \omega ,S_{\omega }y,T_{\omega }x\right)$, which is generated by the two product transformations Θ and Λ.

Let $\pi_{E}:H\to E$, $\pi_{G}:H\to G$ be the two natural projections with $\pi_{E}\left(\omega ,y,x\right)=\left(\omega ,x\right)$, $\pi_{G}\left(\omega ,y,x\right)=\left(\omega ,y\right)$, respectively. Then $\pi_{E}$ and $\pi_{G}$ are obviously two factor transformations. Let D be the restriction of F×C on G and denote $D_{H}=\pi_{G}^{-1}\left(D\right)=\left\{\left(D\times X\right)\cap H:D\in D\right\}$, $A_{H}=\pi_{E}^{-1}\left(A\right)=\left\{\left(A\times Y\right)\cap H:A\in A\right\}$ and $F_{H}=\left\{\left(F\times Y\times X\right)\cap H:F\in F\right\}$.

For the given the σ−algebra $D_{H}$, the relative entropy of Γ is then defined as

$$h_{\mu }\left(\Gamma |D_{H}\right)=\underset{R}{\sup}h_{\mu }\left(R|D_{H}\right),$$

where R belongs to the set of all finite or countable measurable partitions of H satisfying $H_{\mu }\left(R|D_{H}\right)<\infty$.

We need the following two important Lemmas. The first Lemma shows the upper semi-continuity of the conditional entropy, which can be found in many references, for instance [5,7]. The second one is Lemma 5 in [26], which shows the intrinsic connection relating the relative entropy with the relative tail pressure even in the general additive case.

**Lemma** **2.** *Let $R=\{R_{1},\cdots ,R_{k}\}$ be a finite measurable partition of H. If $m\in P_{P}\left(H\right)$ with $m(\partial R_{i})=0$, 1≤i≤k, where $m\left(\partial R\right)=\int m_{\omega }\left(\partial R\left(\omega \right)\right)dP(\omega )$, then*

$$\underset{\mu \to m}{lim sup}H_{\mu }\left(R|D_{H}\right)\leq H_{m}\left(R|D_{H}\right).$$

**Lemma** **3.** *Let $\mu \in P_{P}\left(H\right)$, $f\in L_{E}^{1}\left(\Omega ,C\left(X\right)\right)$ and R,Q be two finite measurable partitions of E. Then*

$$H_{\mu }\left(\pi_{E}^{-1}R|D_{H}\right)+\int f\circ \pi_{E}d\mu \leq H_{\mu }\left(\pi_{E}^{-1}Q|D_{H}\right)+\int \max_{Q\in Q}\log\sum_{R\in R}e^{\alpha (R(\omega ))}dP.$$

*where $\alpha \left(R\left(\omega \right)\right)=\sup_{x\in R(\omega )\cap Q(\omega )}f\left(\omega ,x\right)$.*

For any given finite measurable partition of the original RDS, we give an inequality relating the relative conditional pressure with subadditive potentials and the relative entropy of the product RDS with respect to invariant measures.

**Proposition** **3.** Let *Γ* be the skew product transformation on H, $\mu \in I_{P}\left(H\right)$ and *Φ* be subadditive with $\Phi^{*}\left(\pi_{E}\mu \right)>-\infty$. If Q is a finite measurable partition of E, then

$$h_{\mu }\left(\Gamma |D_{H}\right)+\Phi^{*}\left(\pi_{E}\mu \right)\leq h_{\mu }\left(\pi_{E}^{-1}Q|D_{H}\right)+\pi_{\Theta }^{\Phi }\left(Q\right).$$

**Proof.**  Let $R=\{R_{1},\cdots ,R_{k}\}$ be a measurable partition of E, $\nu =\pi_{E}\mu$ and ϵ>0. Let $V=\{V_{0},V_{1},\cdots ,V_{k}\}$ be a measurable partition of E such that $H_{\nu }\left(R|V\right)<\epsilon$ and $S=\{V_{0}\cup V_{1},\cdots ,V_{0}\cup V_{k}\}$ be the open random cover of E generated by V (see [26] for details). Denote by δ(ω) the Lebesgue number of the open cover S(ω) for each ω∈Ω.

Fix n∈N. Denote $\alpha \left(V\left(\omega \right)\right)=\sup_{x\in V(\omega )\cap Q(\omega )}f_{n}\left(\omega ,x\right),$ where $Q\left(\omega \right)\in Q^{\left(n\right)}\left(\omega \right)$ and $V\left(\omega \right)\in V^{\left(n\right)}\left(\omega \right)$. Choose one point x(V(ω)) in $\bar{V(\omega )\cap Q(\omega )}$ with $f_{n}\left(\omega ,x\left(V\left(\omega \right)\right)\right)=\alpha \left(V\left(\omega \right)\right)$. For each pair of elements $V_{i}\left(\omega \right),V_{j}\left(\omega \right)$ in V(ω), $d(x\left(V_{i}\left(\omega \right)\right),x\left(V_{j}\left(\omega \right)\right))<\delta \left(\omega \right)$ implies that $V_{i}\left(\omega \right)$ and $V_{j}\left(\omega \right)$ are in the same element of S(ω). Hence for each $V\left(\omega \right)\in V^{\left(n\right)}\left(\omega \right)$, there exists at most $2^{n}$ elements $V^{\prime }\left(\omega \right)$ of $V^{\left(n\right)}\left(\omega \right)$ satisfying

$$d_{\delta ,n}^{\omega }\left(V\left(\omega \right),V^{\prime }\left(\omega \right)\right)=\max_{0\leq i<n}\left\{d\left(T_{\omega }^{i}x\left(V\left(\omega \right)\right),T_{\omega }^{i}x\left(V^{\prime }\left(\omega \right)\right)\right){\left(\delta \left(\vartheta^{i}\omega \right)\right)}^{-1}\right\}<1.$$

For each ω, an (n,δ(ω))-separated set $E_{Q}\left(\omega \right)$ satisfying the inequality

(1) $$\sum_{V\in V^{\left(n\right)}}e^{\alpha (V(\omega ))}\leq 2^{n}\sum_{y\in E_{Q}\left(\omega \right)}e^{f_{n}\left(\omega ,y\right)}$$

can be easily constructed in Q(ω) as follows. Choose the first point $x(V^{1}\left(\omega \right))$ with $f_{n}\left(\omega ,x\left(V^{1}\left(\omega \right)\right)\right)=\max_{V\in V^{\left(n\right)}}\alpha \left(V\left(\omega \right)\right),$ the second point $x(V^{2}\left(\omega \right))$ with

$$f_{n}\left(\omega ,x\left(V^{2}\left(\omega \right)\right)\right)=\max_{\begin{array}{c} V\in V^{\left(n\right)}\\ d_{\delta ,n}^{\omega }\left(x\left(V\left(\omega \right)\right),x\left(V^{1}\left(\omega \right)\right)\right)>1 \end{array}}\alpha \left(V\left(\omega \right)\right).$$

Choose the *m*th point $x(V^{m}\left(\omega \right))$ such that

$$f_{n}\left(\omega ,x\left(V^{m}\left(\omega \right)\right)\right)=\max_{\begin{array}{c} V\in V^{\left(n\right)}\\ d_{\delta ,n}^{\omega }\left(x\left(V\left(\omega \right)\right),x\left(V^{i}\left(\omega \right)\right)\right)>1,\;\;1\leq i<m \end{array}}\alpha \left(V\left(\omega \right)\right).$$

The process will cease at some finite step *l* since $V^{\left(n\right)}$ is finite. Let $E_{Q}\left(\omega \right)=\{x\left(V^{1}\left(\omega \right)\right),\cdots ,$$x(V^{l}\left(\omega \right))\}$. $E_{Q}\left(\omega \right)$ is obviously an (n,δ(ω))-separated set and at most $2^{n}$ elements of $V^{\left(n\right)}\left(\omega \right)$ are deleted for each step. The inequality (1) holds.

It follows from Lemma 3 that

$$\begin{array}{cc} \; & \frac{1}{n}H_{\mu }\left(\pi_{E}^{-1}V^{\left(n\right)}|D_{H}\right)+\frac{1}{n}\int f_{n}\circ \pi_{E}d\mu \\ \leq & \frac{1}{n}H_{\mu }\left(\pi_{E}^{-1}Q^{\left(n\right)}|D_{H}\right)+\frac{1}{n}\int \max_{Q\in Q^{\left(n\right)}}\log\sum_{y\in E_{Q}\left(\omega \right)}e^{f_{n}\left(\omega ,y\right)}dP+\log2. \end{array}$$

Let U={U}∈U(E) be an open random cover of E satisfying diam(U(ω))<δ(ω). Since each $U\left(\omega \right)\in U^{\left(n\right)}\left(\omega \right)$ cannot contain two or above elements in $E_{Q}\left(\omega \right)$, then

$$\max_{Q\in Q^{\left(n\right)}}\sum_{y\in E_{Q}\left(\omega \right)}e^{S_{n}f\left(\omega ,y\right)}\leq P_{\Theta }^{\Phi }\left(\omega ,n,U,Q\right).$$

Since $\Theta \pi_{E}=\pi_{E}\Gamma$ and $\Phi^{*}\left(\pi_{E}\mu \right)>-\infty$, then

$$\begin{array}{c} h_{\mu }\left(\pi_{E}^{-1}V|D_{H}\right)+\Phi^{*}\left(\pi_{E}\mu \right)\leq h_{\mu }\left(\pi_{E}^{-1}Q|D_{H}\right)+\pi_{\Theta }^{\Phi }\left(Q\right)+\log2. \end{array}$$

Since

$$\begin{array}{c} h_{\mu }\left(\pi_{E}^{-1}R|D_{H}\right)\leq h_{\mu }\left(\pi_{E}^{-1}P|D_{H}\right)+h_{\nu }\left(R|V\right), \end{array}$$

then

$$\begin{array}{c} h_{\mu }\left(\pi_{E}^{-1}R|D_{H}\right)+\Phi^{*}\left(\pi_{E}\mu \right)\leq h_{\mu }\left(\pi_{E}^{-1}Q|D_{H}\right)+\pi_{\Theta }^{\Phi }\left(Q\right)+\log2+\epsilon . \end{array}$$

Let $R_{1}≺\cdots ≺R_{n}≺\cdots$ be a refine sequence of finite measurable partitions such that ${⋁}_{i=1}^{\infty }R_{n}=A$, then the inequality

(2) $$h_{\mu }\left(\Gamma |D_{H}\right)++\Phi^{*}\left(\pi_{E}\mu \right)\leq h_{\mu }\left(\pi_{E}^{-1}Q|D_{H}\right)+\pi_{\Theta }^{\Phi }\left(Q\right)+\log2+\epsilon$$

follows from Lemma 1.6 in Reference [33].

Observe that

(3) $$h_{\mu ,\Gamma^{{}^{m}}}\left(\overset{m-1}{\underset{i=0}{⋁}}{\left(\Gamma^{i}\right)}^{-1}\pi_{E}^{-1}Q|D_{H}\right)=mh_{\mu }\left(\pi_{E}^{-1}Q|D_{H}\right),$$

where $h_{\mu ,\Gamma^{{}^{m}}}\left(\eta |D_{H}\right)$ is the usual relative entropy of $\Gamma^{m}$*w.r.t.* the partition η.

For each m∈N, by Lemma 1.4 in Reference [33],

(4) $$h_{\mu }\left(\Gamma^{m}|D_{H}\right)=mh_{\mu }\left(\Gamma |D_{H}\right).$$

Using $\Gamma^{m}$, $\Theta^{m}$, $Q^{\left(m\right)}$ and $\Phi^{m}$ in the inequality (2), by the above equalities (3), (4) and the power rules in Proposition 1, one can easily get

$$h_{\mu }\left(\Gamma |D_{H}\right)++\Phi^{*}\left(\pi_{E}\mu \right)\leq h_{\mu }\left(\pi_{E}^{-1}Q|D_{H}\right)+\pi_{\Theta }^{\Phi }\left(Q\right),$$

which completes the proof. □

The following theorem describes the variational inequality between the relative tail pressure with subadditive potentials, the defect of upper semi-continuity of the relative entropy function and Lyapunov exponent with subadditive potentials with respect to invariant measures.

**Theorem** **1.** Let *Γ* be the skew product transformation on H, $m\in I_{P}\left(H\right)$. For subadditive potentials *Φ* with $\Phi^{*}\left(\pi_{E}m\right)>-\infty$, one has $h_{m}^{*}\left(\Gamma |D_{H}\right)+\Phi^{*}\left(\pi_{E}m\right)\leq \pi_{\Theta }^{*}\left(\Phi \right)$.

**Proof.**  Let $\nu =\pi_{E}m$ and Q∈P(E) be finite. Let R be a refine finite measurable partition of Q with ν(∂R)=0 for each element R∈R. Then by Proposition 3,

$$\begin{array}{cc} h_{\mu }\left(\Gamma |D_{H}\right)+\Phi^{*}\left(\pi_{E}\mu \right)\; & \leq h_{\mu }\left(\pi_{E}^{-1}R|D_{H}\right)+\pi_{\Theta }^{\Phi }\left(R\right)\\ \; & \leq \frac{1}{n}H_{\mu }\left(\overset{n-1}{\underset{i=0}{⋁}}{\left(\Gamma^{i}\right)}^{-1}\pi_{E}^{-1}R|D_{H}\right)+\pi_{\Theta }^{\Phi }\left(Q\right), \end{array}$$

for each $\mu \in I_{P}\left(H\right)$ and n∈N. By Lemma 2, the upper semi-continuity of the conditional entropy implies that

$$\begin{array}{c} \underset{\mu \to m}{lim sup}h_{\mu }\left(\Gamma |D_{H}\right)+\Phi^{*}\left(\pi_{E}m\right)\leq \frac{1}{n}H_{m}\left(\overset{n-1}{\underset{i=0}{⋁}}{\left(\Gamma^{i}\right)}^{-1}\pi_{E}^{-1}R|D_{H}\right)+\pi_{\Theta }^{\Phi }\left(Q\right). \end{array}$$

Then

$$\underset{\mu \to m}{lim sup}h_{\mu }\left(\Gamma |D_{H}\right)+\Phi^{*}\left(\pi_{E}m\right)\leq h_{m}\left(\Gamma |D_{H}\right)+\pi_{\Theta }^{\Phi }\left(Q\right).$$

By the arbitrariness of the partition Q, we have $h_{m}^{*}\left(\Gamma |D_{H}\right)+\Phi^{*}\left(\pi_{E}m\right)\leq \pi_{\Theta }^{*}\left(\Phi \right)$. □

## 5. Variational Principle for Relative Tail Pressure

In this section we investigate the variational principle between the defect of upper semi-continuity of the relative entropy function of the self-product RDSs and the relative tail pressure with subadditive potentials of the original RDS.

Denote by $E^{\left(2\right)}=\left\{\left(\omega ,x,y\right):x,y\in E_{\omega }\right\}$ and $A_{E^{\left(2\right)}}=\left\{\left(A\times X\right)\cap E^{\left(2\right)}:A\in F\times B\right\}$. Let $\Theta^{\left(2\right)}:E^{\left(2\right)}\to E^{\left(2\right)}$ with $\Theta^{\left(2\right)}\left(\omega ,x,y\right)=\left(\vartheta \omega ,T_{\omega }x,T_{\omega }y\right)$ be the skew product transformation on $E^{\left(2\right)}$. Let $\pi_{E_{i}}$ be the natural projection from $E^{\left(2\right)}$ to $E_{i}$ with $\pi_{E_{i}}\left(\omega ,x_{1},x_{2}\right)=\left(\omega ,x_{i}\right)$, i=1, 2, where $E_{1}=E_{2}=E$.

We will use the following Lemma [36] in the proof of Proposition 4. It is a random version of the result presented by Cao et al. [4].

**Lemma** **4.** *Let ${\left\{\mu_{n}\right\}}_{n=1}^{\infty }$ be a sequence probability measures in $P_{P}\left(E\right)$, where $\mu_{n}=\frac{1}{n}\sum_{i=0}^{n-1}\Theta^{i}\nu_{n}$ and ${\left\{\nu_{n}\right\}}_{n=1}^{\infty }\subset P_{P}\left(E\right)$. Suppose that $\left\{n_{i}\right\}$ is a subsequence of N with $\mu_{n_{i}}\to \mu$ in $I_{P}\left(E\right)$. Then for each k∈N,*

(5) $$\underset{i\to \infty }{lim sup}\frac{1}{n_{i}}\int f_{n_{i}}\left(\omega ,x\right)\;d\nu_{n_{i}}\leq \frac{1}{k}\int f_{k}\;d\mu .$$

Moreover, $\Phi^{*}\left(\mu \right)$ is an upper bound of the left limit superior.

For any given open random cover of the original RDS, the following construction of a maximal invariant measure sets up a relationship between the relative conditional pressure with subadditive potentials and the relative entropy of the self-product RDSs, which is essential for the argument of the variational principle.

**Proposition** **4.** *Let *Θ* be the skew product transformation on E, *Φ* be subadditive and U∈U(E) with $U=\{U_{1},U_{2},\cdots ,U_{k}\}$. Then there exists some $\mu_{U}\in I_{P}\left(E^{\left(2\right)}\right)$ satisfying*

*(i)*  $h_{\mu_{U}}\left(\Theta^{\left(2\right)}|A_{E^{\left(2\right)}}\right)+\Phi^{*}\left(\pi_{E}\mu_{U}\right)\geq \pi_{\Theta }^{\Phi }\left(U\right)-\frac{1}{k},$
*(ii)*  *the support of $\mu_{U}$ is on $\overset{k}{\underset{j=1}{⋃}}\left\{\left(\omega ,x,y\right)\in E^{\left(2\right)}:x,y\in \bar{U_{j}}\left(\omega \right)\right\}$.*

**Proof.**  Choose some V∈U(E) with $V=\{V_{1},V_{2},\cdots ,V_{l}\}$ such that $\pi_{\Theta }^{\Phi }\left(V,U\right)\geq \pi_{\Theta }^{\Phi }\left(U\right)-\frac{1}{k}$.

Fix n∈N and ω∈Ω. Let U(ω) be an element in $U^{\left(n\right)}\left(\omega \right)$ with $P_{\Theta }^{\Phi }\left(\omega ,n,V,U\right)=P_{\Theta }^{\Phi }\left(\omega ,n,V,U\right)$ and choose one point x∈U(ω). Let η(ω) be the Lebesgue number of the open cover $\left\{V_{1}\left(\omega \right),\cdots ,V_{l}\left(\omega \right)\right\}$. Let $\delta \left(\omega \right)<\frac{\eta (\omega )}{2}$. There exists a maximal (n,δ)-separated subset $E_{n}\left(\omega \right)$ with $U\left(\omega \right)\subset {⋃}_{y\in E_{n}\left(\omega \right)}B_{y}\left(\omega ,n,\delta \right)$ in U(ω), where $B_{y}\left(\omega ,n,\delta \right)={⋂}_{i=0}^{n-1}{\left(T_{\omega }^{i}\right)}^{-1}B\left(T_{\omega }^{i}y,\delta \left(\vartheta^{i}\omega \right)\right)$. Let

$$\tau_{V,n}\left(\omega \right)=\sup\left\{|f_{n}\left(\omega ,x\right)-f_{n}\left(\omega ,y\right)|:d\left(x,y\right)<\mathrm{diam}V^{\left(n\right)}\left(\omega \right)\right\}.$$

Notice that $B_{y}\left(\omega ,n,\delta \right)$ is the subset of some element of $V^{\left(n\right)}\left(\omega \right)$. It follows that

$$\sum_{V\in V^{\left(n\right)}}\underset{x\in V(\omega )\cap U(\omega )}{\sup}\exp\left(f_{n}\left(\omega ,x\right)-\tau_{V,n}\left(\omega \right)\right)\leq \sum_{y\in E_{n}\left(\omega \right)}\exp f_{n}\left(\omega ,y\right),$$

and we have

$$P_{\Theta }^{\Phi }\left(\omega ,n,V,U\right)\cdot \exp\left(-\tau_{V,n}\left(\omega \right)\right)\leq \sum_{y\in E_{n}\left(\omega \right)}\exp f_{n}\left(\omega ,y\right).$$

Let $\sigma^{\left(n\right)}$ be the probability measures of $E^{\left(2\right)}$ such that their disintegrations satisfying

$$\sigma_{\omega }^{\left(n\right)}=\frac{\sum_{z\in E_{n}\left(\omega \right)}\exp\left(f_{n}\circ \pi_{E_{2}}\left(\omega ,x,z\right)\right)\delta_{\left(\omega ,x,z\right)}}{\sum_{y\in E_{n}\left(\omega \right)}\exp\left(f_{n}\circ \pi_{E_{2}}\left(\omega ,x,y\right)\right)}$$

with $d\sigma^{\left(n\right)}\left(\omega ,x,y\right)=d\sigma_{\omega }^{\left(n\right)}\left(x,y\right)dP\left(\omega \right)$. Denote

$$\mu^{\left(n\right)}=\frac{1}{n}\sum_{i=0}^{n-1}{\left(\Theta^{\left(2\right)}\right)}^{i}\sigma^{\left(n\right)}.$$

It follows from Theorem 1.5.8 in Reference [32] that the Krylov-Bogolyubov procedure for continuous bundle RDS guarantees that one can choose a subsequence $\left\{n_{j}\right\}$ of N such that $\mu^{\left(n_{j}\right)}$ converges towards some $\mu_{U}\in I_{P}\left(E^{\left(2\right)}\right)$.

We now show that $\mu_{U}$ satisfies the properties (i) and (ii).

For the first proposition, let $\nu =\pi_{E_{2}}\mu_{U}$. Let R be a finite partition of E into measurable subsets with ν(∂R)=0 and diamR(ω)<δ(ω). Denote $\zeta^{\left(n\right)}={⋁}_{i=0}^{n-1}{\left(\Theta^{\left(2\right)}\right)}^{-i}\pi_{E_{2}}^{-1}R$. Then $\zeta^{\left(n\right)}=\pi_{E_{2}}^{-1}R^{\left(n\right)}$ since $\pi_{E_{2}}\Theta^{\left(2\right)}=\Theta \pi_{E_{2}}$. Let $\zeta^{\left(n\right)}=\left\{C\right\}$.

Let $\pi_{X_{1}}^{-1}B\left(\omega \right)=\left\{\left(B\times X_{2}\right)\cap E_{\omega }^{\left(2\right)}:B\in B\right\}$, where $X_{1}=X_{2}=X$ and $\pi_{X_{1}}$ is the natural projection. It is abbreviated as $\pi_{X_{1}}^{-1}B$ for convenience in the sequel.

Since different elements of $E_{n}\left(\omega \right)$ belong to different elements of $R^{\left(n\right)}\left(\omega \right)$,

(6) $$E\left(1_{C(\omega )}|\pi_{X_{1}}^{-1}B\right)\left(x,y\right)=\sigma_{\omega }^{\left(n\right)}\left(C\left(\omega \right)\right).$$

It is not hard to verify that

$$\begin{array}{cc} \; & H_{\sigma_{\omega }^{\left(n\right)}}\left(\xi^{\left(n\right)}\left(\omega \right)\right)+\int f_{n}\circ \pi_{E_{2}}d\sigma_{\omega }^{\left(n\right)}\\ = & \int \sum_{D\left(\omega \right)\in \xi^{\left(n\right)}\left(\omega \right)}-E\left(1_{D(\omega )}|\pi_{X_{1}}B\right)\log E\left(1_{D(\omega )}|\pi_{X_{1}}B\right)d\sigma_{\omega }^{\left(n\right)}+\int f_{n}\circ \pi_{E_{2}}d\sigma_{\omega }^{\left(n\right)}\\ \; & =\log\sum_{y\in E_{n}\left(\omega \right)}\exp f_{n}\left(\omega ,y\right). \end{array}$$

Notice that

$$E\left(1_{C}|A_{E^{\left(2\right)}}\right)\left(\omega ,x,y\right)=E\left(1_{C(\omega )}|\pi_{X_{1}}B\right)\left(x,y\right)\;\;P-a.s..$$

Then

(7) $$\begin{array}{cc} \; & H_{\sigma^{\left(n\right)}}\left(\zeta^{\left(n\right)}|A_{E^{\left(2\right)}}\right)+\int f_{n}\circ \pi_{E_{2}}d\sigma^{\left(n\right)}\\ = & \int \log\sum_{y\in E_{n}\left(\omega \right)}\exp f_{n}\left(\omega ,y\right)dP\geq \int \log P_{\Theta }^{\Phi }\left(\omega ,n,V,U\right)-\tau_{V,n}\left(\omega \right)dP. \end{array}$$

For each *j* with 0≤j<m<n, the section (0,n−1) can be separated into $[\frac{n}{m}]-2$ subsection (j,j+m−1),⋯,(j+km,j+(k+1)m−1), ⋯ and no more than 3m other positive integers. Then

$$\begin{array}{cc} \; & H_{\sigma^{\left(n\right)}}\left(\zeta^{\left(n\right)}|A_{E^{\left(2\right)}}\right)+\int f_{n}\circ \pi_{E_{2}}d\sigma^{\left(n\right)}\\ \leq & \sum_{k=0}^{[\frac{n}{m}]-2}H_{\sigma^{\left(n\right)}}\left(\overset{j+(k+1)m-1}{\underset{i=j+km}{⋁}}{\left(\Theta^{\left(2\right)}\right)}^{-i}\pi_{E_{2}}^{-1}R|A_{E^{\left(2\right)}}\right)+\int f_{n}\circ \pi_{E_{2}}d\sigma^{\left(n\right)}+3m\log\;q\\ \leq & \sum_{k=0}^{[\frac{n}{m}]-2}H_{{\left(\Theta^{\left(2\right)}\right)}^{j+km}\sigma^{\left(n\right)}}\left(\zeta^{\left(m\right)}|A_{E^{\left(2\right)}}\right)+\int f_{n}\circ \pi_{E_{2}}d\sigma^{\left(n\right)}+3m\log q. \end{array}$$

Since the entropy function $H_{\left(\cdot \right)}$ is concave, then by summing over all *j*, 0≤j<m we have

$$\begin{array}{cc} \; & mH_{\sigma^{\left(n\right)}}\left(\zeta^{\left(n\right)}|A_{E^{\left(2\right)}}\right)+m\int f_{n}\circ \pi_{E_{2}}d\sigma^{\left(n\right)}\\ \leq & \sum_{k=0}^{n-1}H_{{\left(\Theta^{\left(2\right)}\right)}^{k}\sigma^{\left(n\right)}}\left(\zeta^{\left(m\right)}|A_{E^{\left(2\right)}}\right)+m\int f_{n}\circ \pi_{E_{2}}d\sigma^{\left(n\right)}+3m^{2}\log\;q\\ \leq & nH_{\mu^{\left(n\right)}}\left(\zeta^{\left(m\right)}|A_{E^{\left(2\right)}}\right)+m\int f_{n}\circ \pi_{E_{2}}d\sigma^{\left(n\right)}+3m^{2}\log q. \end{array}$$

It follows the inequality (7) that

$$\begin{array}{cc} \; & \frac{1}{m}H_{\mu^{\left(n\right)}}\left(\zeta^{\left(m\right)}|A_{E^{\left(2\right)}}\right)+\frac{1}{n}\int f_{n}\circ \pi_{E_{2}}d\sigma^{\left(n\right)}\\ \; & \geq \frac{1}{n}\int \log P_{\Theta }^{\Phi }\left(\omega ,n,V,U\right)-\tau_{V,n}\left(\omega \right)dP-\frac{3m}{n}\log q. \end{array}$$

Considering the selected subsequence $\left\{n_{j}\right\}$, by Lemmas 2 and 4, we have

$$\begin{array}{cc} \frac{1}{m}H_{\mu_{U}}\left(\zeta^{\left(m\right)}|A_{E^{\left(2\right)}}\right)+\Phi^{*}\left(\pi_{E_{2}}\mu_{U}\right)\; & \geq \pi_{\Theta }^{\Phi }\left(V,U\right)-\lim_{j\to \infty }\frac{1}{n_{j}}\int \tau_{V,n}dP\\ \; & \geq \pi_{\Theta }^{\Phi }\left(U\right)-\frac{1}{k}-\lim_{j\to \infty }\frac{1}{n_{j}}\int \tau_{V,n}dP. \end{array}$$

By taking m→∞, we have

$$h_{\mu_{U}}\left(\pi_{E_{2}}^{-1}R|A_{E^{\left(2\right)}}\right)+\Phi^{*}\left(\pi_{E_{2}}\mu_{U}\right)\geq \pi_{\Theta }^{\Phi }\left(U\right)-\frac{1}{k}-\lim_{j\to \infty }\frac{1}{n_{j}}\int \tau_{V,n}dP.$$

Choose a refine sequence of $V_{1}≺\cdots ≺V_{n}≺\cdots \in U\left(E\right)$ with $\pi_{\Theta }^{\Phi }\left(V,U\right)\geq \pi_{\Theta }^{\Phi }\left(U\right)-\frac{1}{k}$, and a refine sequence $R_{1}≺\cdots ≺R_{n}≺\cdots$ with ${⋁}_{n=1}^{\infty }R_{n}=A$, where each $R_{n}$ is a finite measurable partition. Using Lemma l.6 in Reference [33] we have

$$h_{\mu_{U}}\left(\Theta^{\left(2\right)}|A_{E^{\left(2\right)}}\right)+\Phi^{*}\left(\pi_{E_{2}}\mu_{U}\right)\geq \pi_{\Theta }^{\Phi }\left(U\right)-\frac{1}{k},$$

and the property (i) holds.

For the second proposition, we omit the argument since it is very similar to that of Proposition 4.2 in Reference [26] and we complete the proof. □

We now show that the relative tail pressure with subadditive potentials of the original RDS could be reached by the defect of the upper semi-continuity of the relative entropy together with the Lyapunov exponent of the subadditive potentials with respect to some invariant measure of the self-product RDS.

**Proposition** **5.** Let *Θ* be a skew product transformation on E and *Φ* be subadditive with $\pi_{\Theta }^{*}\left(\Phi \right)>-\infty$. There exists some $m\in I_{P}\left(E^{\left(2\right)}\right)$ with its support on $\left\{\left(\omega ,x,x\right)\in E^{\left(2\right)}:x\in E_{\omega }\right\}$ and $\Phi^{*}\left(\pi_{E}m\right)>-\infty$ such that $h_{m}^{*}\left(\Theta^{\left(2\right)}|A_{E^{\left(2\right)}}\right)+\Phi^{*}\left(\pi_{E}m\right)=\pi_{\Theta }^{*}\left(\Phi \right)$.

**Proof.**  Let $U_{1}≺\cdots ≺U_{n}≺\cdots \in U\left(E\right)$. Denote by $U_{n}={\left\{U_{j}^{\left(n\right)}\right\}}_{j=1}^{k_{n}}$. By Proposition 4, for each n∈N, we can find some $\mu_{n}\in I_{P}\left(E^{\left(2\right)}\right)$ with its support on ${⋃}_{j=1}^{k_{n}}\left\{\left(\omega ,x,y\right):x,y\in \bar{U_{j}^{\left(n\right)}}\left(\omega \right)\right\}$ and satisfying the inequality

$$h_{\mu_{n}}\left(\Theta^{\left(2\right)}|A_{E^{\left(2\right)}}\right)+\Phi^{*}\left(\pi_{E}\mu_{n}\right)\geq \pi_{\Theta }^{\Phi }\left(U_{n}\right)-\frac{1}{k_{n}}.$$

By ([7] Lemma 2.1) the set of the limit points of the sequence of $\mu_{n}$ is contained in $I_{P}\left(E^{\left(2\right)}\right)$. Pick some limit point *m* with $\lim_{l\to \infty }\mu_{l}=m$ for some subsequence $n_{l}$ of {n}, then

$$\begin{array}{c} \underset{\mu \to m}{lim sup}\left(h_{\mu }\left(\Theta^{\left(2\right)}|A_{E^{\left(2\right)}}\right)+\Phi^{*}\left(\pi_{E}\mu \right)\right)\geq \underset{n}{\inf}\pi_{\Theta }^{\Phi }\left(U_{n}\right)=\pi_{\Theta }^{*}\left(\Phi \right). \end{array}$$

Notice that

$$\mathrm{supp}m=\overset{\infty }{\underset{l=1}{⋂}}\overset{k_{n_{l}}}{\underset{j=1}{⋃}}\left\{\left(\omega ,x,y\right):x,y\in \bar{U_{j}^{\left(n_{l}\right)}}\left(\omega \right)\right\},$$

and the sequence of open random covers $\left\{U^{n_{l}}\right\}$ is refine, one has

$$\mathrm{supp}m=\{\left(\omega ,x,x\right)\in E^{\left(2\right)}:x\in E_{\omega }\}.$$

Let $\xi =\{\xi_{1},\cdots ,\xi_{k}\}$ be any finite measurable partition of E, obviously,

$$m\left(\pi_{E_{1}}^{-1}\xi_{i}\right)=m\left(\pi_{E_{1}}^{-1}\xi_{i}\cap \mathrm{supp}m\right)=m\left(\pi_{E_{2}}^{-1}\xi_{i}\right)),\;1\leq i\leq k,$$

then the two partitions $\pi_{E_{1}}^{-1}\xi$ and $\pi_{E_{2}}^{-1}\xi$ are the same except zero-measure sets. Notice that $E\left(1_{\pi_{E_{1}}^{-1}\xi_{i}}|A_{E^{\left(2\right)}}\right)=1_{\pi_{E_{1}}^{-1}\xi_{i}}$P−a.s. for each *i*, 1≤i≤k. One has

$$H_{m}\left(\pi_{E_{2}}^{-1}\xi |A_{E^{\left(2\right)}}\right)=H_{m}\left(\pi_{E_{1}}^{-1}\xi |A_{E^{\left(2\right)}}\right)=0,$$

and $h_{m}\left(\Theta^{\left(2\right)}|A_{E^{\left(2\right)}}\right)=0$. Hence

$$\begin{array}{c} h_{m}^{*}\left(\Theta^{\left(2\right)}|A_{E^{\left(2\right)}}\right)+\Phi^{*}\left(\pi_{E}m\right)\geq \pi_{\Theta }^{*}\left(\Phi \right). \end{array}$$

Since $\pi_{\Theta }^{*}\left(\Phi \right)>-\infty$, then $\Phi^{*}\left(\pi_{E}m\right)>-\infty$. By Theorem 1, the result holds. □

It follows directly from Theorem 1 and Proposition 5 that the desired variational principle holds.

**Theorem** **2.** Let *Θ* be a skew product transformation on E and *Φ* be subadditive with $\pi_{\Theta }^{*}\left(\Phi \right)>-\infty$. Then

$$\max\left\{h_{\mu }^{*}\left(\Theta^{\left(2\right)}|A_{E^{\left(2\right)}}\right)+\Phi^{*}\left(\pi_{E}\mu \right):\mu \in I_{P}\left(E^{\left(2\right)}\right),\Phi^{*}\left(\pi_{E}\mu \right)>-\infty \right\}=\pi_{\Theta }^{*}\left(\Phi \right).$$

Let A be σ-algebra generated by the restriction of the product σ-algebra F×B on E and denote $A_{G}=\left\{\pi^{-1}A:A\in A\right\}$.

**Definition** **2.** *A skew product transformation *Λ* is called a principal extension of the skew product transformation *Θ* if the relative entropy $h_{m}\left(\Lambda |A_{G}\right)$ vanishes for any measure m in $I_{P}\left(G\right)$.*

The following theorem shows that the relative tail pressure with subadditive potentials is invariant under principal extensions. The proof is similar to Theorem 4.3 in Reference [26] and we omit it.

**Theorem** **3.** Let Θ,Λ be two skew product transformations and *Φ* be subadditive with $\pi_{\Theta }^{*}\left(\Phi \right)>-\infty$. If *Λ* is a principal extension of *Θ*, then $\pi_{\Lambda }^{*}\left(\Phi \circ \pi \right)=\pi_{\Theta }^{*}\left(\Phi \right)$, where π is the factor transformation between *Λ* and *Θ* and $\Phi \circ \pi ={\left\{f_{n}\circ \pi \right\}}_{n=1}^{\infty }$.
